# Work stress, anthropometry, lung function, blood pressure, and blood-based biomarkers: a cross-sectional study of 43,593 French men and women

**DOI:** 10.1038/s41598-017-07508-x

**Published:** 2017-08-24

**Authors:** Linda L. Magnusson Hanson, Hugo Westerlund, Marcel Goldberg, Marie Zins, Jussi Vahtera, Naja Hulvej Rod, Sari Stenholm, Andrew Steptoe, Mika Kivimäki

**Affiliations:** 1 Stress Research Institute, Stockholm University, Stockholm, Sweden; 2 Department of Clinical Neuroscience, Division of Insurance Medicine, Karolinska Institutet, Stockholm, Sweden; 3 Inserm, Population-based Epidemiologic Cohorts Unit-UMS 011, Villejuif, France; 4 Paris Descartes University, Paris, France; 5 Department of Public Health, University of Turku, and Turku University Hospital, Turku, Finland; 6 Turku University Hospital, Turku, Finland; 7 Department of Public Health, Copenhagen University, Copenhagen, Denmark; 8 Department of Behavioural Science and Health, University College London, London, UK; 9 Clinicum, Faculty of Medicine, University of Helsinki, Helsinki, Finland; 10 Department of Epidemiology and Public Health, University College London, London, UK

## Abstract

Work stress is a risk factor for cardio-metabolic diseases, but few large-scale studies have examined the clinical profile of individuals with work stress. To address this limitation, we conducted a cross-sectional study including 43,593 working adults from a French population-based sample aged 18–72 years (the CONSTANCES cohort). According to the Effort-Reward Imbalance model, work stress was defined as an imbalance between perceived high efforts and low rewards at work. A standardized health examination included measures of anthropometry, lung function, blood pressure and standard blood-based biomarkers. Linear regression analyses before and after multivariable adjustment for age, socioeconomic status, depressive symptoms, health-related behaviours, and chronic conditions showed that work stress was associated with higher BMI, waist circumference, waist-hip ratio, alanine transaminase, white blood cell count and lower high-density lipoprotein cholesterol in men, and with higher BMI and white blood cell count in women (differences 0.03–0.06 standard deviations, P < 0.05 between individuals with and without work stress). No robust associations were observed with lung function, haemoglobin, creatinine, glucose levels or resting blood pressure measures. This indicates that work stress is associated altered metabolic profile, increased systemic inflammation, and, in men, poorer liver function, which is a marker of high alcohol consumption.

## Introduction

Research on stress and cardiovascular disease has a long history. At the beginning of the 20th century, Sir William Osler, the “father” of modern medicine, suggested that a major cause of myocardial infarction was the “wear and tear of life”^1^. Systematic research on stress at work began in the late 1970s and early 1980s when Robert Karasek launched the Demand-control model postulating that work stress results from a combination of high psychological job demands and low job control^2–4^. More recent work stress theories have broadened the concept beyond these proximal job task characteristics to embrace organizational factors, labour market arrangements, and personal characteristics^5^. The Effort-reward imbalance model, for example, proposes that an imbalance between high efforts and low reward at work is a common source of work-related stress^6^. High efforts may originate from high demands or obligations at work, but also from a personal motivational pattern characterised by a very high commitment to work. Low rewards, in return, can include material (e.g. low salary), social (e.g. poor job security, few promotion prospects) and psychological aspects (e.g. low appreciation, lack of positive feedback). The Effort-reward imbalance is considered as a general conceptualisation of work stress which applies across different occupational settings and types of work.

The 2016 European Guidelines for Prevention of Cardiovascular Disease recommend that work stress is considered as a prevention target, particularly for individuals with high overall cardiovascular disease risk^7^. This recommendation is supported by many prospective cohort studies that have linked work stress (resulting either from high demands and low job control or an imbalance between efforts and rewards) to increased occurrence of e.g. diabetes, coronary heart disease and stroke^8–12^. However, while the associations with these disease endpoints appears established, surprisingly few large-scale studies have sought to clarify potential physiological underpinnings for these associations. To increase opportunities for identifying early stress-related changes, investigations that comprehensively characterize the physiological risk profile of individuals with work stress are warranted.

To address this limitation, we conducted a cross-sectional study of over 40,000 employed men and women examining anthropometric characteristics, lung function, blood pressure and blood-based biomarkers that can routinely be assessed in primary care. We hypothesized that work stress, defined by effort-reward imbalance at work, is associated with adverse adiposity, metabolic, respiratory and inflammatory biomarker levels that characterize an adverse cardiovascular profile. Accordingly, our aim was to quantify differences in those characteristics and biomarkers between individuals with and without work stress. Given the important differences in cardiovascular disease risk between men and women, we stratified the analyses by sex. As socioeconomic status, lifestyle, depression and chronic diseases may be associated with work stress, these factors were taken into account in multivariable adjustments.

## Results

Table 1 presents the main characteristics of the study sample. The sample consisted of a relatively equal proportion of men and women at similar ages (48% men and 52% women, with a mean age of 44.0 and 43.3 years of age, respectively). More men than women had high social position, were smokers, physically active on a regular basis, and had risky alcohol consumption. A higher proportion of men also had night work and physically difficult work whereas sleep disturbances and depressive symptoms were on average more common in women. With regard to chronic conditions, women were less likely to have cardiovascular disease, but more likely to have endocrine disorders and cancer.

**Table 1:** Characteristics of the study population, by sex and work stress.

Characteristic		Men						Women					
		All		No work stress		Work stress		All		No work stress		Work stress	
		N/mean	%/SD	N/mean	%/SD	N/mean	%/SD	N/mean	%/SD	N/mean	%/SD	N/mean	%/SD
Age, y	Range 18.5 to 72.5	44.0	10.6	43.8	11.3	44.3	9.8	43.3	10.8	42.7	11.4	43.9	10.2
Social position^a^	Low	6091	30.9	3385	33.4	2706	30.8	8174	38.2	4271	43.1	3903	36.9
	Intermediate	4836	24.5	2547	25.2	2289	26.0	6643	31.0	3076	31.2	3567	33.8
	High	7993	40.5	4194	41.4	3799	43.2	5655	26.4	2555	25.8	3100	29.3
Current job^b^	Labourer semi-skilled worker	968	5.0	585	5.7	383	4.3	311	1.5	199	2.0	112	1.0
	Skilled or highly skilled worker, shop technician	2716	14.2	1427	13.8	1289	14.5	642	3.1	331	3.3	311	2.9
	Supervisor	1557	8.1	742	7.2	815	9.2	1105	5.3	457	4.6	648	6.0
	Chief Executive Officer deputy CEO	620	3.2	304	3.0	316	3.5	248	1.2	106	1.1	142	1.3
	Technician, draughtsman, sales representative	1610	8.4	822	8.0	788	8.9	805	3.9	360	3.6	445	4.2
	Primary school teacher, social worker, nurse public service	1005	5.2	550	5.3	455	5.1	3160	15.2	1491	14.9	1669	15.6
	Engineer executive	6034	31.4	3070	29.8	2964	33.3	3865	18.6	1711	17.1	2154	20.1
	Teacher public service	2160	11.3	1318	12.8	842	9.5	3104	15.0	1480	14.8	1624	15.2
	Office or commercial employee, duty officer, nursing auxiliary, child minder public service	1872	9.8	1084	10.5	788	8.9	6564	31.7	3342	33.4	3222	30.1
	Other	657	3.4	406	3.9	251	2.8	936	4.5	541	5.4	395	3.7
Night work^c^	No	13907	68.8	7790	71.3	6117	65.8	17806	81.3	8889	82.8	8917	79.8
	Yes	6316	31.2	3132	28.9	3184	34.2	4100	18.7	1845	17.2	2255	20.2
Physically	No	14856	73.5	8360	76.5	6496	69.8	18112	82.7	9104	84.8	9008	80.6
difficult work^d^	Yes	5367	26.5	2562	23.5	2805	30.2	3794	17.3	1630	15.2	2164	19.4
Smoking^e^	Current	5775	27.6	3052	27.1	2723	28.2	5852	26.0	2837	25.8	3015	26.3
	Former	6384	30. 5	3391	30.1	2993	31.0	5939	26.4	2826	25.7	3113	27.1
	Never	8759	41.9	4835	42.9	3924	40.7	10684	47.5	5345	48.6	5339	46.6
Alcohol	Abstinent	514	2.6	303	2.79	211	2.28	890	4.2	495	4.8	395	3.7
Consumption^f^	Neither abuse nor dependence	13857	68.9	7522	69.3	6335	68.3	16425	77.8	8016	77.4	8409	78.1
	Abuse	4215	20.9	2215	20.4	2000	21.6	3155	14.9	1549	15.0	1606	14.9
	Dependent	1539	7.7	811	7.5	728	7.8	651	3.1	295	2.9	356	3.3
Physical activity^g^	Regular sports activity less than 2 hours per week	13445	64.3	6951	61.7	6494	67.4	16081	71.7	7676	69.8	8405	73.5
	Regular sports activity for 2 hours or more per week	7454	35.7	4319	38.3	3135	32.6	6351	28.3	3325	30.2	3026	26.5
Sleep	No	15546	76.0	8927	80.9	6619	70.2	14341	65.3	7717	71.8	6624	59.1
Disturbances^h^	Yes	4916	24.0	2106	19.1	2810	29.8	7626	34.7	3039	28.2	4587	40.9
Depressive symptoms^i^	CES-D score (0–60)	9.3	7.6	7.7	6.5	11.2	8.4	11.9	9.2	9.8	7.9	14.0	9.8
Chronic disease	Cardiovascular disease	1058	5.0	563	5.0	495	5.1	1068	4.7	517	4.7	551	4.8
	Endocrine disorders	2232	10.6	1124	9.9	1108	11.4	3705	16.4	1704	15.4	2001	17.4
	Respiratory disease	2200	10.5	1086	9.6	1114	11.5	2297	10.2	1051	9.5	1246	10.8
	Osteoarticular arthritis	1975	9.4	982	8.7	993	10.3	2018	8.9	887	8.0	1131	9.8
	Cancer	256	1.2	144	1.3	112	1.2	844	3.7	397	3.6	447	3.9
	Any of the above	6392	30.4	3250	28.7	3142	32.5	8026	35.5	3734	33.7	4292	37.3

^a^2448 (5.6%) had missing data and 1753 (4.0%) claimed that they had never worked or had unknown socioeconomic status. ^b^3654 (8.4%) had multiple grades or missing data. ^c^1464 (3.4%) had missing data. ^d^1464 (3.4%) had missing data. ^e^200 (0.5%) had missing data. ^f^2347 (5.4%) had missing data. ^g^262 (0.6%) had missing data. ^h^1164 (2.7%) had missing data ^i^1233 (2.8%) had missing data.

Table 1 also shows that in total, 48.6% of the sample experienced work stress, 46.1% of men and 51.0% of women. Depressive symptoms, sleep disturbance, physical difficult work, night work, and physical inactivity were more common among participants with work stress. With regard to the measures of anthropometry, lung function, blood pressure and standard blood-based biomarkers, there were high correlations (r > 0.70) between BMI and waist circumference, waist circumference and waist hip ratio, FVC and FEV, and blood pressure measures.

**Figure 1:**
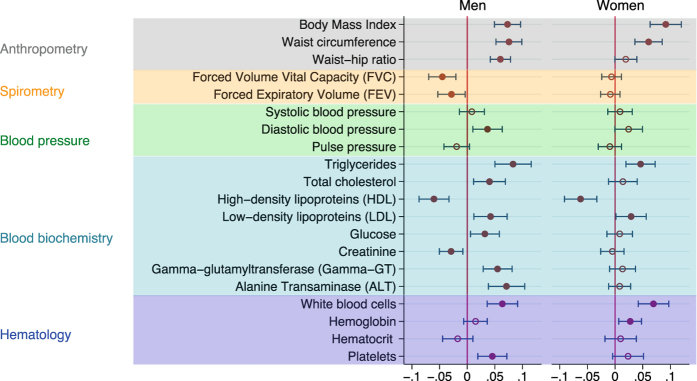
Results of regression analyses of work stress (ERI ratio >1) and measures from the health examinations, while adjusting for age and socioeconomic position. Coefficients estimate the difference on the standardized scale between individuals with work stress compared to those with no work stress.

**Figure 2:**
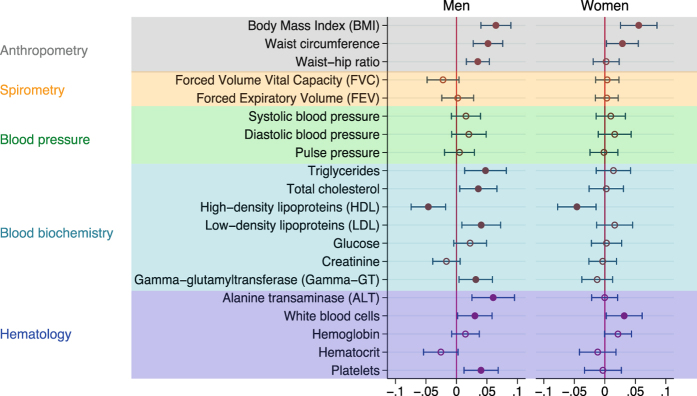
Results of regression analyses of work stress (ERI ratio >1) and measures from the health examinations, while adjusting for age, socioeconomic position, depressive symptoms, health-related behaviours (physical inactivity, smoking and alcohol consumption) and chronic conditions (cardiovascular disease, endocrine disorders, respiratory disease, osteoarticular arthritis, and cancer). Coefficients estimate the difference on the standardized scale between individuals with work stress compared to those with no work stress.

Figure 1 shows that work stress was associated with higher BMI both in men (β = 0.07 indicating a difference of 0.07 standard deviations) and women (β = 0.09; P < 0.001), when adjusting for age and socioeconomic status. In both men and women, work stress was also associated with a higher waist circumference (β = 0.08 and 0.06, respectively; P < 0.001). Work stress was associated with higher waist-hip ratio among men only (β = 0.06; P < 0.001). As presented in Fig. 2 all these associations remained after further adjustment for depressive symptoms, health-related behaviours and chronic diseases. No robust associations were noted between work stress and blood pressure measurements: systolic and diastolic blood pressure and pulse pressure. With regard to spirometry, the analyses showed a relationship between work stress and lower FVC (β = −0.05; P < 0.001) and FEV (β = −0.03 SD; P = 0.02) among men, in the models adjusting for age and socioeconomic status (Fig. 1), but these associations were lost after further adjustments (Fig. 2). Work stress was not associated with FVC and FEV in women.

Figures 1 and 2 also show that analyses of blood biochemistry suggested some stress-related differences in blood lipids, especially for male employees. Triglycerides (β = 0.08; P < 0.001), total cholesterol (β = 0.04; P = 0.006), and LDL cholesterol (β = 0.04; P = 0.006) were all higher among men with work stress as compared to those without, whereas HDL cholesterol was lower (β = −0.06; P < 0.001) (Fig. 1). These relationships remained after adjustments (Fig. 2). In women with work stress compared to women without work stress, triglycerides (β = 0.05; P = 0.001) and LDL cholesterol (β = 0.03; P = 0.037) were higher and HDL cholesterol was lower (β = −0.06; P =< 0.001) after adjustment for age and socioeconomic status, but only the association with HDL remained after further adjusting for depressive symptoms. In men, but not in women, work stress was associated with higher blood glucose when adjusting for age, socioeconomic status and depressive symptoms (β = 0.04; P = 0.01), as well with higher gamma GT, ALT and platelets before and after serial adjustments (β = 0.03; P = 0.023, β = 0.06; P = 0.001, β = 0.04; P = 0.005, respectively, in the fully adjusted model) (Figs 1 and 2). Work stress was associated with higher white blood cell count among both men (β = 0.03; P = 0.036) and women (β = 0.03; P = 0.034) after multivariable adjustment (Fig. 2).

Supportive information presenting the results of linear regression analyses with adjustments for age, socioeconomic position, depressive symptoms, health-related behaviours, and chronic conditions by sex are given in Supplementary Tables S1 and S2.

### Sensitivity analysis

**Figure 3:**
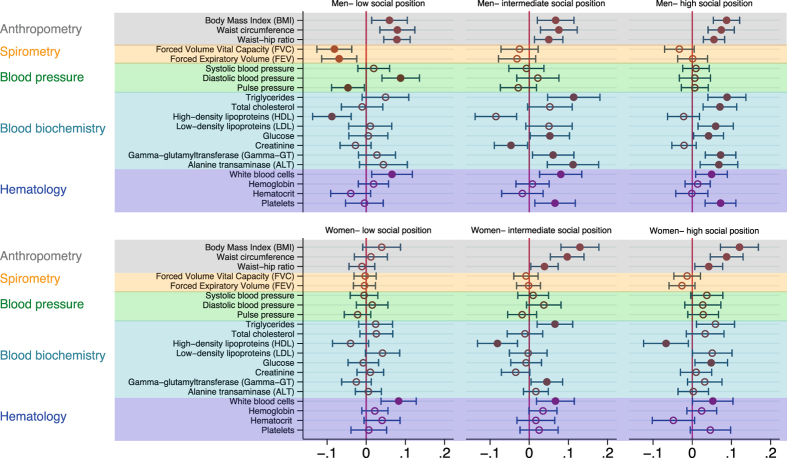
(**a**) Results of regression analyses of work stress (ERI ratio >1) and measures from the health examinations among men, while adjusting for age, divided by social position. Coefficients estimate the difference on the standardized scale between individuals with work stress compared to those with no work stress. (**b**) Results of regression analyses of work stress (ERI ratio >1) and measures from the health examinations among women, while adjusting for age, divided by social position. Coefficients estimate the difference on the standardized scale between individuals with work stress compared to those with no work stress.

The results were similar when considering full time employees only (Supplementary Figure S2). Overall the age-adjusted associations were also similar in people with low, intermediate and high social position. A notable exception was that work stress was related to higher diastolic blood pressure (and hence also with a decreased pulse pressure) among men with low social position but not among men with intermediate or high social position. In contrast, stress-related alterations in lipids were more marked among men with higher social position, except for HDL cholesterol. Furthermore, the association between work stress and triglycerides and HDL cholesterol was slightly more pronounced among women with higher social position (Fig. 3a and b). In sensitivity analyses using the effort-reward ratio as a continuous variable, higher work stress was associated with the same biomarker measures as in the main analyses, but among men also with lower values on the lung function measures, higher diastolic blood pressure and higher glucose (Supplementary Tables S3 and S4). The estimates of association remained similar when adjusting for night work, physically difficult work, and sleep disturbances in addition to age, socioeconomic position, health-related behaviours, depressive symptoms, and chronic conditions (Supplementary Table S5).

## Discussion

Findings from over 43,000 men and women suggest that work stress is associated with altered metabolic profile, in particular adverse adiposity and blood lipid parameters, as well as with increased systemic inflammation as indicated by elevated white cell count. In men but not in women, work stress was additionally linked to measures of poorer liver function. These findings were not attributable to other lifestyle factors, depressive symptoms or chronic conditions, and the associations with metabolic and inflammatory factors were evident at each level of socioeconomic status. No robust associations were observed with lung function, haemoglobin, glucose levels or blood pressure measures, including pulse pressure.

Our findings show that work stress in terms of effort-reward imbalance is linked to altered biomarker levels across multiple systems that affect the risk of diabetes and cardiovascular disease. After taking into account all the main and sensitivity analyses, the most robust associations with work stress among men were those of BMI, waist circumference, waist-hip ratio, HDL, and white blood cell count. Among women, the most robust associations were observed with BMI and white blood cell count.

Our results are in agreement with the hypothesis that work stress is a risk factor for obesity^13–16^ and metabolic syndrome^15, 17, 18^, which may involve adiposity, as well as reduced glucose tolerance, dyslipidaemia, or elevated blood pressure. The present findings provide counter-evidence to studies that have failed to observe an association with regard to adiposity and diabetes^19^. These inconsistencies may partly stem from the potentially heterogeneous effects of stress, as work stress appear to induce weight gain in some people, but be related to weight loss and loss of appetite in others^16^.

Our finding that work stress may be related to dyslipidaemia, especially low HDL cholesterol, is in line with at least two previous studies^13, 20^, although this is not a universal observation^21–23^. Increased levels of white blood cells in stressed individuals may indicate that work stress also affects the immune system by increasing systemic inflammation, a factor that increases the risk of plaque rupture. This finding accords with reviews on work stress and immunity which concluded that effort-reward imbalance is related to reduced immune function^24, 25^. Given that Mendelian randomization studies have confirmed a causal role of inflammation in the aetiology of coronary heart disease^26^, our findings on work stress and increased white cell count may point to a potential pathway that link stress and coronary heart disease. Inflammation is also known to induce depressive symptoms^27^, and work stress is a risk factor for depression^28–30^. However, in the present study, confounding or mediation by depression is unlikely to completely explain the association between work stress and increased inflammation because the association remained after adjustment for depressive symptoms.

Our observation on the association of work stress with adverse liver function indicators in men is novel. This is likely to reflect increased alcohol consumption among stressed men. Work stress, as indicated by extensive working hours, has been associated with increased risky drinking behaviour^31^. Work stress assessed by effort-reward imbalance has also been suggested to increase the risk for alcohol dependence among men^32^, pointing to a potential indirect behavioural pathway between work stress and cardiovascular diseases via increased alcohol consumption as an unhealthy coping strategy to relieve feelings of stress. However, with cross-sectional data we cannot exclude the possibility that heavy drinking may also increase the likelihood of experiencing stress at work. In multivariable adjusted analyses, the relationship between work stress and gamma GT and ALT remained after adjustment for alcohol consumption, but this is likely to reflect imprecise measurement of alcohol consumption using self-reports.

Episodic stress may induce temporary increases in blood pressure and heart rate^33^, but no consistent association between chronic work stress and resting blood pressure was apparent in the present study (although a slight tendency for elevated blood pressure in stressed men was observed). In a recent review of the literature on work stress, as indicated by effort-reward imbalance, Gilbert-Quimet *et al*.^34^ concluded that previous evidence on the relationship with blood pressure is conflicting, but that the findings have been more consistent for men^34^. More recently, the IPD-Work consortium using raw individual-level data from 8 European cohort studies found no consistent association of work stress, as indicated by job strain, with systolic or diastolic blood pressure or hypertension^35^, whereas a smaller study reported that imbalance between efforts and rewards was associated with increased systolic blood pressure in women^36^. More research seems warranted to clarify the potential role of work stress for blood pressure changes, its effect modifiers and the possibility that blood pressure differences become evident only with ambulatory or real-life measurements. We did not observe a robust association between work stress and high blood glucose whereas there was an association among men with higher platelets, a biomarker that assesses the tendency for blood coagulation. Previous evidence on these blood biomarkers is scarce and mixed including both null results^20^ and positive findings^37, 38^.

This study has its strengths and limitations. A major strength is the large population-based sample allowing precise estimation of the relationship between work stress and a large number of anthropometric, functional and blood-based measures. The study population was diverse including persons living and working in various settings, from large cities to small villages in different regions of France, and with a broad range of socioeconomic statuses and trades, supporting the generalizability of our findings. An obvious limitation is the low response rate, which raises the question of selection bias, although exposure-outcome relationships may not differ between subjects who are included and those who are not^39^. Moreover, we measured work stress with efforts and rewards and did not cover other aspects of work stress, such as high demands, low job control or long working hours. An effort-reward ratio indicating that efforts exceed rewards represented work stress in accordance with the standard definition of effort-reward imbalance, but a sensitivity analysis of the continuous ERI-ratio, as suggested by Siegrist *et al*.^40^, also supported the main findings. Due to the cross-sectional nature of the study we cannot draw conclusions about cause and effect relationships and it cannot be ruled out that, for instance, adiposity could influence the experience of effort-reward imbalance. Furthermore, more detailed measures of inflammatory activity would have been desirable as well as measures of ambulatory blood pressure which might be superior to blood pressure measures obtained in the clinic in capturing stress-related changes in vascular functioning^41^. No data were available from ethnic groups preventing us to examine possible ethnic or racial differences in work stress and physiological parameters.

Despite these limitations, the present evidence lends support for a link of work stress with increased adiposity and systemic inflammation, altered metabolic profile, and, in men but not in women, poorer liver function. Given the large sample size, the present study is also important in suggesting that there are no strong associations between effort-reward imbalance, lung function, haemoglobin, glucose levels, and resting blood pressure measures.

## Methods

### Study population

We included participants of the French CONSTANCES cohort, a general-purpose population-based cohort intended to serve as an open epidemiological research infrastructure^42, 43^. CONSTANCES was started in 2012 with the aim of collecting data from a total of 200,000 individuals over a 5-year period. The cohort is made up of French adults aged 18–69 years at inception who were affiliated to the General Health Insurance Fund in France (about 85% of the general population). People insured by the General Health Insurance Fund include salaried workers, professionally active or retired and their families. Agricultural workers and self-employed were not included in the study. Participants were invited to respond to questionnaires and to a health examination in one of 22 selected health screening centres across principal regions of France (7% response rate). Those invited were randomly selected individuals affiliated to the General Health Insurance Fund in the selected catchment areas with stratification according to unequal response probabilities, based on experiences from previous surveys involving invitations to health screening^44^. At the time of this study, data on work stress were available for 43,593 employed participants, the study sample of the present analyses (Figure S1).

### Sociodemographic characteristics, work characteristics, lifestyle and health status

We obtained information on the participants’ sex, age (divided into 5-year bands for analyses), and socioeconomic status from self-administered questionnaires. We categorized socioeconomic status into three groups: low (e.g., office or commercial employee, child minder, manual worker), intermediate (e.g., teachers, nurses, social worker, technicians, foremen, supervisors), and high (e.g., executives, engineers, physicians). Current job grade/qualification was further classified into 10 categories: (1) Labourer, semi-skilled worker, (2) Skilled worker, highly skilled worker, shop technician, (3) Supervisor, (4) Chief Executive Officer, deputy CEO, (5) Technician, draughtsman, sales representative, (6) Primary school teacher, social worker, nurse, public service, (7) Engineer, executive, (8) Teacher, public service, (9) Office or commercial employee, duty officer, nursing auxiliary, child minder, public service, and (10) Other. Based on responses to questions on organisational constraints throughout the working life we derived information about current/recent night work (yes or no) and physically difficult work (yes or no). Health-related behaviours, measured using standard questionnaires, were smoking (current, former, or never smoker), physical activity (regular sports activity for 2 hours or more per week or less than 2 hours per week), and alcohol consumption. Alcohol consumption was assessed by means of AUDIT (10 items) and categorized into abstinence; no alcohol abuse nor dependence; and alcohol abuse (AUDIT score 8–12 for men and 7–11 for women) or alcohol dependence (AUDIT score >12 for men and >11 for women)^45, 46^. Four questions were also used to assess sleep problems: difficulties falling asleep, repeated awakening during the night, early awakening, and non-refreshed sleep. Respondents were considered suffering from sleep disturbances if they reported any of the above sleep problems 15 days or more during the past month. Self-reported depressive symptoms were assessed using the Center for Epidemiological Studies Depression Scale (CES-D)^47^. Participants reported whether they had been diagnosed with cardiovascular disease (angina pectoris, myocardial infarction, stroke, lower limb arteritis or other cardiovascular diseases), endocrine disorders (thyroid, diabetes, hypercholesterolemia, hypertriglyceridemia, or other endocrine disorders), respiratory disease (chronic bronchitis or asthma), osteoarticular arthritis (inflammatory arthritis or other osteoarticular disorders), and cancer at any point before the health examination.

### Assessment of work stress

We used a short version of the effort-reward imbalance questionnaire to assess work stress^6, 40^. The effort scale included 3 items (e.g. “I have constant time pressure due to heavy work load” and “Over the past few years my job has become more and more demanding”) and the reward scale included 7 items (e.g., “I receive the respect I deserve from my superior or a respective relevant person”, “My job promotion prospects are poor”, “My job security is poor”, “Considering all my efforts and achievements, my salary/income is adequate”)^40^. The 4 response options for each item ranged from strongly disagree to strongly agree. Based on the respective scale scores we calculated an effort/reward ratio using a correction factor for unequal number of items (mean ratio 1.06, SD 0.45). Values over 1 representing a situation with high efforts in combination with low rewards defined work stress and all other values denoted no work stress^48^.

### Anthropometry, lung function, blood pressure and blood-based biomarkers

The participants were invited to a health examination standardized by means of Standard Operating Procedures (SOPs). As part of the SOPs, distributors of medical devices were asked to comply with international guidelines and CONSTANCES requirements, and participating centres accepted to revise their practices. Quality control was ensured through close monitoring in collaboration with the ClinSearch Company and the Asqualab and Eurocell Associations. The purpose of the standardisation and quality control was to ensure high quality physiological data from multiple sites despite unequal conditions^49^. Nurses doing the health examinations were trained in advance and were blind to the participants’ work stress scores. Weight and height were measured for calculation of body mass index (BMI, weight in kilograms divided by height in meters squared). Waist and hip circumference were measured to assess waist-hip ratio. Blood pressure measurements were taken from each arm (after a 5-minute rest and 2 minutes in between measurements) and one measurement on the reference arm after 1 minute rest. From measurements of systolic and diastolic blood pressure we calculated pulse pressure (systolic minus diastolic blood pressure). To assess lung function, spirometry was performed with 3 measures each of forced volume vital capacity (FVC) and forced expiratory volume (FEV), of which the highest of the 3 measurements was used^50^.

Laboratory tests based on blood samples included measurement of blood sugar level, lipid work-up, liver function tests, blood creatinine levels, and complete blood cell counts. Participants were instructed to fast for 12 hours before the blood test which was performed between 8 AM and 10 AM. Blood sugar level was assessed by blood glucose, and lipids by total cholesterol (TC), high-density lipoproteins (HDL), and triglycerides (TG). Levels of low density lipoproteins (LDL) were also calculated based on values on TC, HDL and TG (TC- HDL-(TG/2.2)). Values for glucose and lipids were considered valid if participants had been fasting for a minimum of 8 hours. Liver function was additionally measured by gamma-glutamyltransferase (gamma GT), and alanine transaminase (ALT), while blood creatinine was used as an indicator of kidney function. Blood biology included counts of white blood cells, haemoglobin, haematocrit (packed cell volume), and platelets.

The biomarker data were first screened for outliers and unreasonable values removed.

### Data analysis

We performed separate linear regression analyses to study the association of work stress with anthropometry and biomarkers. After excluding missing or unreasonable biomarker values, the analytic samples ranged between between n = 31,903 to n = 43,197 depending on the measure (a lower proportion had complete data on spirometry and valid data on lipids). Before analysis, BMI, triglycerides, glucose, creatinine, gamma GT, white blood cell count, and platelet values were logarithmically transformed, while values on haematocrit were squared to decrease skewness. After transformation of the data all biomarkers had skewness ≤2, indicating no substantial departure from the normal distribution^51^. The scores on each of the measures were subsequently standardized (Mean = 0, Standard deviation = 1) to allow comparison between measures.

Analyses were performed for each of the parameters separately stratified by sex. The basic models were adjusted for age. Multivariable adjustment was additionally performed for socioeconomic status, CES-D depressive symptoms, health-related behaviours, and chronic conditions. To further examine the role of socioeconomic position, we stratified analyses by this variable. Sensitivity analyses were performed on a subsample working full time (n = 34,375) and using the entire effort-reward ratio as a continuous variable^40^. In addition, we added to multivariable adjusted models work characteristics, such as night work and physically difficult work, and sleep disturbances.

### Ethical considerations

The CONSTANCES Cohort project has obtained authorization from the French National Data Protection Authority (“Commission nationale de l’informatique et des libertés”) and have been approved by the National Council for Statistical Information, the National Medical Council, and the Institutional Review Board of the National Institute for Medical Research-INSERM. Informed consent was obtained from all participants. The analyses were carried out in accordance with the relevant guidelines and regulations.

### Data availability

The data that support the findings of this study are not publicly available due to legal restrictions, but applications for data access can be submitted in the context of calls for proposals. For more information about how to make use of the CONSTANCES cohort, see http://www.constances.fr/index_EN.php.

## Electronic supplementary material

Supporting tables
