# Supplementary material for: Work stress, anthropometry, lung function, blood pressure, and blood-based biomarkers: a cross-sectional study of 43,593 French men and women
Source: Sci Rep. 2017 Aug 24;7:9282. doi: 10.1038/s41598-017-07508-x (PMC5570902; doi:10.1038/s41598-017-07508-x)
Supplement: Supplementary file 1 — Supporting tables [file 41598_2017_7508_MOESM1_ESM.pdf]

Work stress, anthropometry, lung function, blood pressure, and blood-based biomarkers: a cross-sectional study of 43,593 French men and women

Linda L. Magnusson Hanson[1,*], Hugo Westerlund[1,2], Marcel Goldberg[3,4], Marie Zins[3,4], Jussi Vahtera[5,6], Naja Hulvej Rod[7], Sari Stenholm[5], Andrew Steptoe[8], Mika Kivimäki[19,8]

1. Stress Research Institute, Stockholm University, Sweden
2. Department of Clinical Neuroscience, Division of Insurance Medicine, Karolinska Institutet, Sweden
3. Inserm, Population-based Epidemiologic Cohorts Unit-UMS 011, Villejuif, France
4. Paris Descartes University, Paris, France
5. Department of Public Health, University of Turku, and Turku University Hospital, Finland
6. Turku University Hospital, Finland
7. Department of Public Health, Copenhagen University, Copenhagen, Denmark
8. Clinicum, Faculty of Medicine, University of Helsinki, Helsinki, Finland
9. Department of Epidemiology and Public Health, University College London, London UK

Supplementary table S1. Results from regression analyses on work stress (ERI ratio>1) and measures from the health examinations *among men*, with serial adjustments for social position, health behaviours, chronic diseases, depressive symptoms. Coefficients estimate the difference on the standardized scale between individuals with work stress compared to those with no work stress.

	Model 1				*Model 2*				*Model 3*				*Model 4*				*Model 5*			
	β	*95 % CI*		*P*	*β*	*95 % CI*		*P*	*β*	*95 % CI*		*P*	*β*	*95 % CI*		*P*	*β*	*95 % CI*		*P*
		L	*U*			*L*	*U*			*L*	*U*			*L*	*U*			*L*	*U*	
**Anthropometry**																				
Body Mass Index (BMI)	0.07	0.05	0.09	0.000#	0.07	0.05	0.10	0.000#	0.08	0.05	0.10	0.000#	0.07	0.04	0.09	0.000#	0.06	0.04	0.09	0.000#
Waist circumference	0.07	0.05	0.09	0.000#	0.08	0.05	0.10	0.000#	0.07	0.05	0.10	0.000#	0.06	0.03	0.08	0.000#	0.05	0.03	0.08	0.000#
Waist-hip ratio	0.05	0.04	0.07	0.000#	0.06	0.04	0.08	0.000#	0.05	0.03	0.07	0.000#	0.04	0.02	0.06	0.000#	0.04	0.02	0.05	0.000#
**Spirometry**																				
Forced Volume Vital Capacity (FVC)	-0.04	-0.06	-0.02	0.001#	-0.05	-0.07	-0.02	0.000#	-0.03	-0.06	0.00	0.021	-0.03	-0.05	0.00	0.052	-0.02	-0.05	0.00	0.100
Forced Expiratory Volume (FEV)	-0.03	-0.05	-0.01	0.016	-0.03	-0.05	0.00	0.024	-0.01	-0.03	0.02	0.506	0.00	-0.03	0.02	0.719	0.00	-0.02	0.03	0.875
**Blood pressure**																				
Systolic blood pressure	0.01	-0.01	0.03	0.433	0.01	-0.01	0.03	0.461	0.02	-0.01	0.04	0.158	0.02	-0.01	0.04	0.144	0.02	-0.01	0.04	0.201
Diastolic blood pressure	0.04	0.01	0.06	0.006	0.04	0.01	0.06	0.007	0.03	0.00	0.06	0.029	0.02	-0.01	0.05	0.124	0.02	-0.01	0.05	0.156
Pulse pressure	-0.02	-0.04	0.00	0.127	-0.02	-0.04	0.00	0.105	0.00	-0.03	0.02	0.865	0.01	-0.02	0.03	0.593	0.00	-0.02	0.03	0.691
**Blood biochemistry**																				
Triglycerides	0.08	0.05	0.11	0.000#	0.08	0.05	0.12	0.000#	0.07	0.03	0.10	0.000#	0.05	0.02	0.09	0.003#	0.05	0.01	0.08	0.006
Total cholesterol	0.04	0.02	0.07	0.002#	0.04	0.01	0.07	0.006	0.05	0.02	0.08	0.003	0.03	0.00	0.06	0.030	0.04	0.01	0.07	0.021
High-density lipoproteins (HDL)	-0.06	-0.09	-0.04	0.000#	-0.06	-0.09	-0.03	0.000#	-0.05	-0.08	-0.02	0.000#	-0.05	-0.08	-0.02	0.001#	-0.05	-0.07	-0.02	0.001#
Low-density lipoproteins (LDL)	0.05	0.02	0.08	0.001#	0.04	0.01	0.07	0.006	0.05	0.02	0.08	0.002#	0.04	0.01	0.07	0.019	0.04	0.01	0.07	0.012
Glucose	0.04	0.01	0.06	0.004	0.03	0.01	0.06	0.016	0.04	0.01	0.06	0.010	0.03	0.00	0.06	0.045	0.02	0.00	0.05	0.102
Creatinine	-0.02	-0.04	0.00	0.019	-0.03	-0.05	-0.01	0.007	-0.02	-0.04	0.00	0.124	-0.02	-0.04	0.01	0.138	-0.02	-0.04	0.01	0.153
Gamma-glutamyltransferase (Gamma-GT)	0.05	0.02	0.07	0.000#	0.05	0.03	0.08	0.000#	0.05	0.02	0.08	0.000#	0.04	0.01	0.06	0.011	0.03	0.00	0.06	0.023
Alanine transaminase (ALT)	0.07	0.04	0.10	0.000#	0.07	0.04	0.10	0.000#	0.07	0.04	0.11	0.000#	0.07	0.03	0.10	0.000#	0.06	0.03	0.10	0.001#
**Haematology**																				
White blood cells	0.06	0.03	0.09	0.000#	0.06	0.04	0.09	0.000#	0.04	0.01	0.07	0.006	0.03	0.01	0.06	0.020	0.03	0.00	0.06	0.036
Haemoglobin	0.01	-0.01	0.03	0.176	0.01	-0.01	0.04	0.169	0.02	0.00	0.04	0.087	0.01	-0.01	0.04	0.194	0.01	-0.01	0.04	0.199
Haematocrit	-0.02	-0.05	0.01	0.156	-0.02	-0.04	0.01	0.222	-0.03	-0.06	0.00	0.051	-0.03	-0.05	0.00	0.073	-0.03	-0.05	0.00	0.075
Platelets	0.04	0.02	0.07	0.001#	0.05	0.02	0.07	0.001#	0.04	0.01	0.07	0.004	0.04	0.01	0.07	0.003	0.04	0.01	0.07	0.005

statistically significant after Bonferroni correction for multiple testing

β=regression coefficient, CI=confidence interval, L=lower limit, U=upper limit

Model 1: adjusted for age, Model 2: adjusted for age, socioeconomic position, Model 3: adjusted for age, socioeconomic position, and depressive symptoms, Model 4: adjusted for age, socioeconomic position, depressive symptoms, and health behaviours, Model 5: adjusted for age, socioeconomic position, depressive symptoms, health behaviours and chronic diseases.

Supplementary table S2. Results from regression models on work stress (ERI ratio>1) and measures from the health examinations *among women,* with serial adjustments for social position, health behaviours, chronic diseases, depressive symptoms among. Coefficients estimate the difference on the standardized scale between individuals with work stress compared to those with no work stress.

	Model 1				***Model 2***				***Model 3***				***Model 4***				***Model 5***			
	β	***95% CI***		***P***	***β***	***95% CI***		***P***	***β***	***95% CI***		***P***	***β***	***95% CI***		***P***	***β***	***95% CI***		***P***
		L	***U***			***L***	***U***			***L***	***U***			***L***	***U***			***L***	***U***	
**Anthropometry**																				
Body Mass Index (BMI)	0.07	0.05	0.10	0.000#	0.09	0.06	0.12	0.000#	0.06	0.03	0.09	0.000#	0.06	0.03	0.09	0.000#	0.06	0.03	0.09	0.000#
Waist circumference	0.04	0.02	0.07	0.000#	0.06	0.04	0.09	0.000#	0.04	0.01	0.06	0.005	0.03	0.01	0.06	0.016	0.03	0.00	0.05	0.031
Waist-hip ratio	0.01	-0.01	0.03	0.408	0.02	0.00	0.04	0.056	0.01	-0.01	0.03	0.385	0.00	-0.02	0.03	0.699	0.00	-0.02	0.02	0.845
**Spirometry**																				
Forced Volume Vital Capacity (FVC)	0.00	-0.01	0.02	0.570	-0.01	-0.02	0.01	0.482	0.00	-0.02	0.02	0.915	0.00	-0.02	0.02	0.776	0.00	-0.02	0.02	0.684
Forced Expiratory Volume (FEV)	0.00	-0.01	0.02	0.711	-0.01	-0.03	0.01	0.334	0.00	-0.02	0.02	0.943	0.00	-0.02	0.02	0.884	0.00	-0.02	0.02	0.737
**Blood pressure**																				
Systolic blood pressure	0.00	-0.03	0.02	0.714	0.01	-0.01	0.03	0.428	0.01	-0.01	0.03	0.424	0.01	-0.01	0.03	0.411	0.01	-0.01	0.03	0.427
Diastolic blood pressure	0.01	-0.01	0.04	0.250	0.02	0.00	0.05	0.054	0.02	-0.01	0.04	0.238	0.02	-0.01	0.04	0.225	0.02	-0.01	0.04	0.240
Pulse pressure	-0.02	-0.04	0.00	0.066	-0.01	-0.03	0.01	0.384	0.00	-0.02	0.02	0.901	0.00	-0.02	0.02	0.901	0.00	-0.02	0.02	0.898
**Blood biochemistry**																				
Triglycerides	0.04	0.01	0.06	0.005	0.05	0.02	0.07	0.001#	0.02	0.00	0.05	0.097	0.02	-0.01	0.05	0.241	0.01	-0.01	0.04	0.329
Total cholesterol	0.01	-0.02	0.03	0.506	0.01	-0.01	0.04	0.289	0.01	-0.02	0.04	0.519	0.00	-0.02	0.03	0.813	0.00	-0.03	0.03	0.871
High-density lipoproteins (HDL)	-0.06	-0.08	-0.03	0.000#	-0.06	-0.09	-0.03	0.000#	-0.05	-0.08	-0.02	0.001#	-0.05	-0.08	-0.02	0.003	-0.05	-0.08	-0.01	0.004
Low-density lipoproteins (LDL)	0.02	0.00	0.05	0.093	0.03	0.00	0.06	0.037	0.02	-0.01	0.05	0.119	0.02	-0.01	0.05	0.248	0.02	-0.01	0.05	0.285
Glucose	0.00	-0.02	0.03	0.751	0.01	-0.01	0.03	0.482	0.00	-0.02	0.03	0.704	0.01	-0.02	0.03	0.689	0.00	-0.01	0.03	0.823
Creatinine	0.00	-0.02	0.02	0.957	0.00	-0.03	0.02	0.644	0.00	-0.03	0.02	0.763	0.00	-0.03	0.02	0.738	0.00	-0.03	0.02	0.765
Gamma-glutamyltransferase (Gamma-GT)	0.01	-0.02	0.03	0.604	0.01	-0.01	0.04	0.259	0.00	-0.03	0.02	0.859	-0.01	-0.04	0.01	0.404	-0.01	-0.04	0.01	0.333
Alanine transaminase (ALT)	0.01	-0.01	0.03	0.366	0.01	-0.01	0.03	0.414	0.00	-0.02	0.02	0.952	0.00	-0.02	0.02	0.955	0.00	-0.02	0.02	0.973
**Haematology**																				
White blood cells	0.06	0.03	0.08	0.000#	0.07	0.04	0.10	0.000#	0.04	0.01	0.07	0.003	0.03	0.00	0.06	0.023	0.03	0.00	0.06	0.034
Haemoglobin	0.02	0.00	0.04	0.021	0.03	0.01	0.05	0.009	0.03	0.00	0.05	0.018	0.02	0.00	0.04	0.049	0.02	0.00	0.04	0.054
Haematocrit	0.00	-0.03	0.03	0.999	0.01	-0.02	0.04	0.486	-0.01	-0.04	0.02	0.680	-0.01	-0.04	0.02	0.430	-0.01	-0.04	0.02	0.444
Platelets	0.01	-0.02	0.04	0.539	0.02	0.00	0.05	0.099	0.01	-0.02	0.04	0.447	0.00	-0.03	0.03	0.931	0.00	-0.03	0.03	0.838

statistically significant after Bonferroni correction for multiple testing

β=regression coefficient, CI=confidence interval, L=lower limit, U=upper limit

Model 1: adjusted for age, Model 2: adjusted for age, and socioeconomic position, Model 3: adjusted for age, socioeconomic position, and depressive symptoms, Model 4: adjusted for age, socioeconomic position, depressive symptoms, and health behaviours, Model 5: adjusted for age, socioeconomic position, depressive symptoms, health behaviours and chronic diseases.

Supplementary table S3. Results from regression models on work stress (continuous ERI ratio) and measures from the health examinations *among men*, with serial adjustments for social position, health behaviours, chronic diseases, depressive symptoms. Coefficients estimate the difference on the standardized scale between individuals with work stress compared to those with no work stress.

	Model 1				***Model 2***				***Model 3***				***Model 4***				***Model 5***			
	β	***95 % CI***		***P***	***β***	***95 % CI***		***P***	***β***	***95 % CI***		***P***	***β***	***95 % CI***		***P***	***β***	***95 % CI***		***P***
		L	***U***			***L***	***U***			***L***	***U***			***L***	***U***			***L***	***U***	
**Anthropometry**																				
Body Mass Index (BMI)	0.07	0.05	0.10	0.000#	0.07	0.05	0.10	0.000#	0.08	0.05	0.11	0.000#	0.07	0.04	0.10	0.000#	0.06	0.03	0.09	0.000#
Waist circumference	0.09	0.06	0.11	0.000#	0.09	0.06	0.12	0.000#	0.08	0.05	0.11	0.000#	0.06	0.03	0.09	0.000#	0.06	0.03	0.09	0.000#
Waist-hip ratio	0.08	0.06	0.10	0.000#	0.08	0.06	0.10	0.000#	0.07	0.04	0.09	0.000#	0.05	0.03	0.07	0.000#	0.05	0.02	0.07	0.000#
**Spirometry**																				
Forced Volume Vital Capacity (FVC)	-0.08	-0.11	-0.05	0.000#	-0.08	-0.11	-0.05	0.000#	-0.06	-0.09	-0.03	0.000#	-0.06	-0.09	-0.03	0.000#	-0.06	-0.09	-0.03	0.000#
Forced Expiratory Volume (FEV)	-0.07	-0.10	-0.04	0.000#	-0.07	-0.10	-0.04	0.000#	-0.04	-0.07	-0.01	0.007	-0.04	-0.07	-0.01	0.009	-0.03	-0.06	0.00	0.045
**Blood pressure**																				
Systolic blood pressure	0.01	-0.01	0.04	0.311	0.01	-0.02	0.04	0.447	0.02	-0.01	0.05	0.181	0.02	-0.01	0.05	0.154	0.02	-0.01	0.05	0.183
Diastolic blood pressure	0.06	0.03	0.08	0.000#	0.06	0.03	0.09	0.000#	0.05	0.02	0.08	0.003	0.04	0.01	0.07	0.020	0.04	0.01	0.07	0.023
Pulse pressure	-0.03	-0.05	0.00	0.030	-0.03	-0.06	-0.01	0.011	-0.01	-0.04	0.01	0.313	0.00	-0.03	0.03	0.782	-0.01	-0.03	0.02	0.719
**Blood biochemistry**																				
Triglycerides	0.09	0.05	0.13	0.000#	0.10	0.06	0.13	0.000#	0.07	0.03	0.11	0.001#	0.05	0.01	0.09	0.015	0.05	0.01	0.09	0.026
Total cholesterol	0.05	0.02	0.08	0.002#	0.05	0.02	0.08	0.004	0.06	0.02	0.09	0.002#	0.05	0.01	0.08	0.013	0.05	0.01	0.09	0.008
High-density lipoproteins (HDL)	-0.08	-0.11	-0.05	0.000#	-0.08	-0.11	-0.05	0.000#	-0.07	-0.10	-0.03	0.000#	-0.06	-0.10	-0.03	0.000#	-0.06	-0.09	-0.02	0.001#
Low-density lipoproteins (LDL)	0.06	0.03	0.09	0.001#	0.06	0.02	0.09	0.002#	0.07	0.03	0.10	0.000#	0.06	0.02	0.09	0.004	0.06	0.02	0.10	0.002#
Glucose	0.06	0.03	0.09	0.000#	0.05	0.02	0.08	0.002#	0.05	0.02	0.09	0.001#	0.05	0.02	0.08	0.004	0.05	0.01	0.08	0.006
Creatinine	-0.04	-0.07	-0.02	0.000#	-0.04	-0.07	-0.02	0.001#	-0.02	-0.05	0.00	0.067	-0.02	-0.05	0.00	0.099	-0.02	-0.05	0.01	0.111
Gamma-glutamyltransferase (Gamma-GT)	0.06	0.03	0.09	0.000#	0.07	0.04	0.10	0.000#	0.06	0.03	0.09	0.000#	0.04	0.01	0.08	0.011	0.04	0.01	0.07	0.022
Alanine transaminase (ALT)	0.06	0.02	0.10	0.001#	0.06	0.03	0.10	0.001#	0.07	0.02	0.11	0.002#	0.05	0.01	0.10	0.014	0.05	0.01	0.09	0.025
**Haematology**																				
White blood cells	0.08	0.05	0.12	0.000#	0.08	0.05	0.12	0.000#	0.04	0.01	0.08	0.010	0.04	0.01	0.08	0.017	0.04	0.00	0.07	0.031
Haemoglobin	0.02	-0.01	0.04	0.195	0.02	-0.01	0.04	0.212	0.02	0.00	0.05	0.099	0.02	-0.01	0.04	0.249	0.02	-0.01	0.04	0.250
Haematocrit	0.00	-0.03	0.03	0.943	0.00	-0.03	0.03	0.934	-0.02	-0.05	0.02	0.341	-0.01	-0.05	0.02	0.430	-0.01	-0.05	0.02	0.421
Platelets	0.04	0.01	0.07	0.003	0.05	0.02	0.08	0.001#	0.05	0.01	0.08	0.007	0.05	0.02	0.08	0.003	0.05	0.02	0.08	0.004

statistically significant after Bonferroni correction for multiple testing

β=regression coefficient, CI=confidence interval, L=lower limit, U=upper limit

Model 1: adjusted for age, Model 2: adjusted for age, and socioeconomic position, Model 3: adjusted for age, socioeconomic position, and depressive symptoms, Model 4: adjusted for age, socioeconomic position, depressive symptoms, and health behaviours, Model 5: adjusted for age, socioeconomic position, depressive symptoms, health behaviours and chronic diseases.

Supplementary table S4. Results from regression models on work stress (continuous ERI ratio) and measures from the health examinations *among women*, with serial adjustments for social position, health behaviours, chronic diseases, depressive symptoms. Coefficients estimate the difference on the standardized scale between individuals with work stress compared to those with no work stress.

	Model 1				*Model 2*				*Model 3*				*Model 4*				*Model 5*			
	β	*95 % CI*		*P*	*β*	*95 % CI*		*P*	*β*	*95 % CI*		*P*	*β*	*95 % CI*		*P*	*β*	*95 % CI*		*P*
		L	*U*			*L*	*U*			*L*	*U*			*L*	*U*			*L*	*U*	
**Anthropometry**																				
Body Mass Index (BMI)	0.11	0.08	0.14	0.000#	0.13	0.10	0.17	0.000#	0.10	0.06	0.13	0.000#	0.091	0.057	0.125	0.000#	0.08	0.05	0.12	0.000#
Waist circumference	0.08	0.05	0.10	0.000#	0.10	0.07	0.13	0.000#	0.07	0.04	0.10	0.000#	0.063	0.034	0.093	0.000#	0.06	0.03	0.09	0.000#
Waist-hip ratio	0.02	0.00	0.04	0.044	0.04	0.02	0.06	0.001#	0.02	0.00	0.05	0.050	0.018	-0.006	0.042	0.136	0.01	-0.01	0.04	0.258
**Spirometry**																				
Forced Volume Vital Capacity (FVC)	0.00	-0.02	0.01	0.632	-0.01	-0.03	0.01	0.201	0.00	-0.02	0.02	0.973	0.003	-0.019	0.025	0.774	0.01	-0.02	0.03	0.614
Forced Expiratory Volume (FEV)	-0.01	-0.03	0.00	0.122	-0.02	-0.04	-0.01	0.012	-0.01	-0.03	0.01	0.265	-0.009	-0.031	0.012	0.400	-0.01	-0.03	0.02	0.591
**Blood pressure**																				
Systolic blood pressure	0.00	-0.02	0.03	0.784	0.02	-0.01	0.04	0.138	0.02	-0.01	0.05	0.121	0.023	-0.004	0.050	0.098	0.02	-0.01	0.05	0.120
Diastolic blood pressure	0.02	0.00	0.05	0.090	0.03	0.01	0.06	0.016	0.02	-0.01	0.05	0.132	0.024	-0.006	0.055	0.115	0.02	-0.01	0.05	0.140
Pulse pressure	-0.02	-0.04	0.01	0.178	0.00	-0.03	0.02	0.789	0.01	-0.02	0.03	0.465	0.011	-0.015	0.037	0.415	0.01	-0.02	0.04	0.444
**Blood biochemistry**																				
Triglycerides	0.07	0.04	0.10	0.000#	0.08	0.05	0.11	0.000#	0.05	0.02	0.08	0.001#	0.045	0.013	0.076	0.006	0.04	0.01	0.07	0.015
Total cholesterol	0.01	-0.02	0.04	0.438	0.01	-0.01	0.04	0.355	0.01	-0.02	0.04	0.646	-0.001	-0.032	0.031	0.975	0.00	-0.03	0.03	0.904
High-density lipoproteins (HDL)	-0.09	-0.12	-0.06	0.000#	-0.10	-0.13	-0.06	0.000#	-0.08	-0.12	-0.05	0.000#	-0.082	-0.117	-0.047	0.000#	-0.08	-0.11	-0.04	0.000#
Low-density lipoproteins (LDL)	0.03	0.01	0.06	0.017	0.04	0.01	0.07	0.011	0.03	0.00	0.06	0.047	0.025	-0.008	0.058	0.140	0.02	-0.01	0.06	0.174
Glucose	0.00	-0.02	0.02	0.999	0.01	-0.02	0.03	0.633	0.00	-0.02	0.03	0.883	0.002	-0.026	0.030	0.890	0.00	-0.03	0.03	0.856
Creatinine	-0.01	-0.03	0.01	0.358	-0.01	-0.04	0.01	0.244	-0.01	-0.03	0.02	0.473	-0.010	-0.035	0.016	0.454	-0.01	-0.03	0.02	0.479
Gamma-glutamyltransferase (Gamma-GT)	0.01	-0.01	0.03	0.453	0.02	-0.01	0.04	0.195	-0.01	-0.03	0.02	0.696	-0.009	-0.038	0.019	0.521	-0.01	-0.04	0.02	0.389
Alanine transaminase (ALT)	0.00	-0.02	0.02	0.892	0.00	-0.02	0.02	0.776	-0.01	-0.03	0.02	0.605	-0.005	-0.029	0.019	0.705	-0.01	-0.03	0.02	0.584
**Haematology**																				
White blood cells	0.07	0.04	0.10	0.000#	0.08	0.05	0.11	0.000#	0.05	0.02	0.08	0.003	0.039	0.006	0.072	0.020	0.04	0.00	0.07	0.037
Haemoglobin	0.02	0.00	0.04	0.029	0.02	0.00	0.05	0.046	0.02	0.00	0.04	0.090	0.014	-0.011	0.039	0.265	0.01	-0.01	0.04	0.300
Haematocrit	0.00	-0.03	0.03	0.817	0.01	-0.02	0.04	0.518	-0.01	-0.04	0.03	0.740	-0.015	-0.048	0.019	0.391	-0.01	-0.05	0.02	0.412
Platelets	0.02	-0.01	0.05	0.127	0.04	0.01	0.07	0.018	0.02	-0.01	0.05	0.186	0.016	-0.018	0.050	0.365	0.01	-0.02	0.05	0.456

statistically significant after Bonferroni correction for multiple testing

β=regression coefficient, CI=confidence interval, L=lower limit, U=upper limit

Model 1: adjusted for age, Model 2: adjusted for age, and socioeconomic position, Model 3: adjusted for age, socioeconomic position, and depressive symptoms, Model 4: adjusted for age, socioeconomic position, depressive symptoms, and health behaviours, Model 5: adjusted for age, socioeconomic position, depressive symptoms, health behaviours and chronic diseases.

Supplementary table S5. Results from regression analyses on work stress (ERI ratio>1) and measures from the health examinations, with adjustments for age, social position, night work, physically difficult work, health behaviours, chronic diseases, depressive symptoms, and sleep disturbances. Coefficients estimate the difference on the standardized scale between individuals with work stress compared to those with no work stress.

	Men				*Women*			
	β	*95 % CI*		*P*	*β*	*95 % CI*		*P*
		L	*U*			*L*	*U*	
**Anthropometry**								
Body Mass Index (BMI)	0.06	0.03	0.08	0.000#	0.04	0.01	0.07	0.003
Waist circumference	0.04	0.02	0.07	0.000#	0.02	0.00	0.05	0.096
Waist-hip ratio	0.03	0.01	0.05	0.002#	0.00	-0.02	0.02	0.986
**Spirometry**								
Forced Volume Vital Capacity (FVC)	-0.02	-0.04	0.01	0.185	0.00	-0.02	0.02	0.664
Forced Expiratory Volume (FEV)	0.00	-0.02	0.03	0.735	0.00	-0.02	0.02	0.686
**Blood pressure**								
Systolic blood pressure	0.01	-0.01	0.04	0.297	0.01	-0.02	0.03	0.486
Diastolic blood pressure	0.01	-0.01	0.04	0.327	0.02	-0.01	0.04	0.252
Pulse pressure	0.01	-0.02	0.03	0.615	-0.00	-0.03	0.02	0.814
**Blood biochemistry**								
Triglycerides	0.04	0.01	0.07	0.024	0.01	-0.02	0.04	0.395
Total cholesterol	0.03	-0.00	0.06	0.057	-0.00	-0.03	0.03	0.900
High-density lipoproteins (HDL)	-0.04	-0.07	-0.01	0.004	-0.04	-0.08	-0.01	0.008
Low-density lipoproteins (LDL)	0.04	0.00	0.07	0.027	0.01	-0.02	0.04	0.497
Glucose	0.02	-0.01	0.05	0.143	0.00	-0.03	0.03	0.993
Creatinine	-0.01	-0.04	0.01	0.218	-0.00	-0.02	0.02	0.958
Gamma-glutamyltransferase (Gamma-GT)	0.03	0.00	0.06	0.038	-0.01	0.04	0.01	0.267
Alanine transaminase (ALT)	0.06	0.02	0.09	0.002#	-0.00	-0.03	0.02	0.757
**Haematology**								
White blood cells	0.03	-0.00	0.06	0.062	0.03	-0.00	0.06	0.065
Haemoglobin	0.01	-0.01	0.04	0.289	0.02	-0.00	0.04	0.064
Haematocrit	-0.03	-0.06	0.00	0.067	-0.01	-0.04	0.02	0.447
Platelets	0.04	0.01	0.07	0.004	-0.00	-0.03	0.03	0.847

statistically significant after Bonferroni correction for multiple testing

β=regression coefficient, CI=confidence interval, L=lower limit, U=upper limit

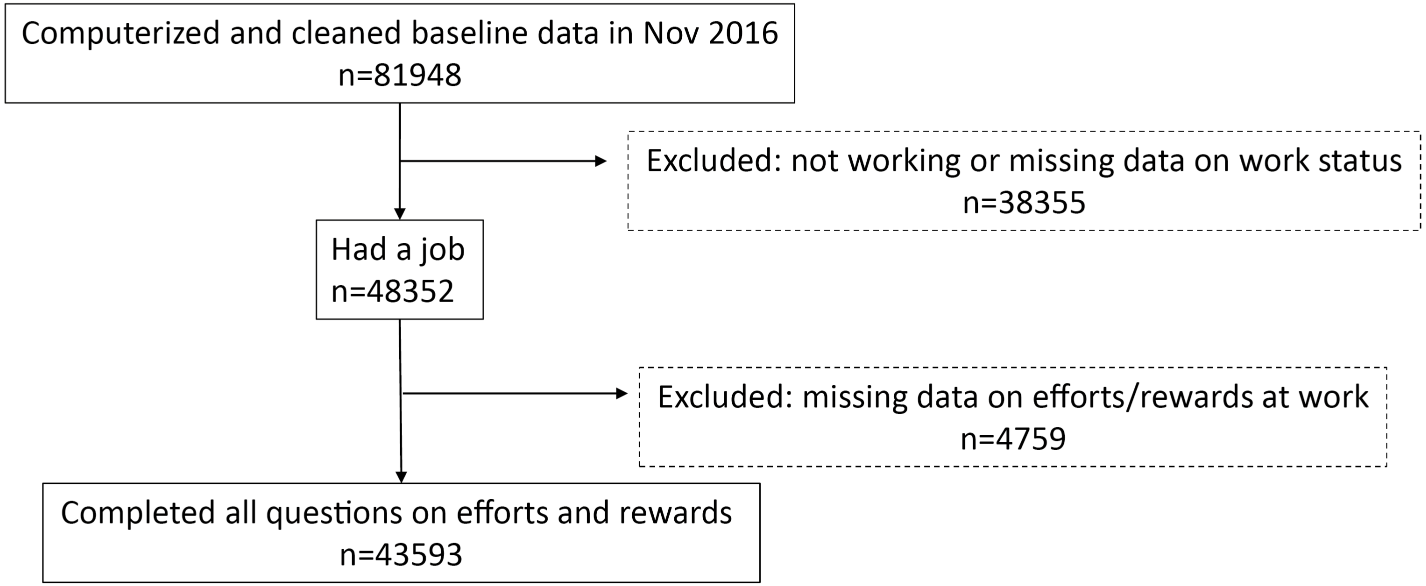

Supplementary figure S1. Flowchart

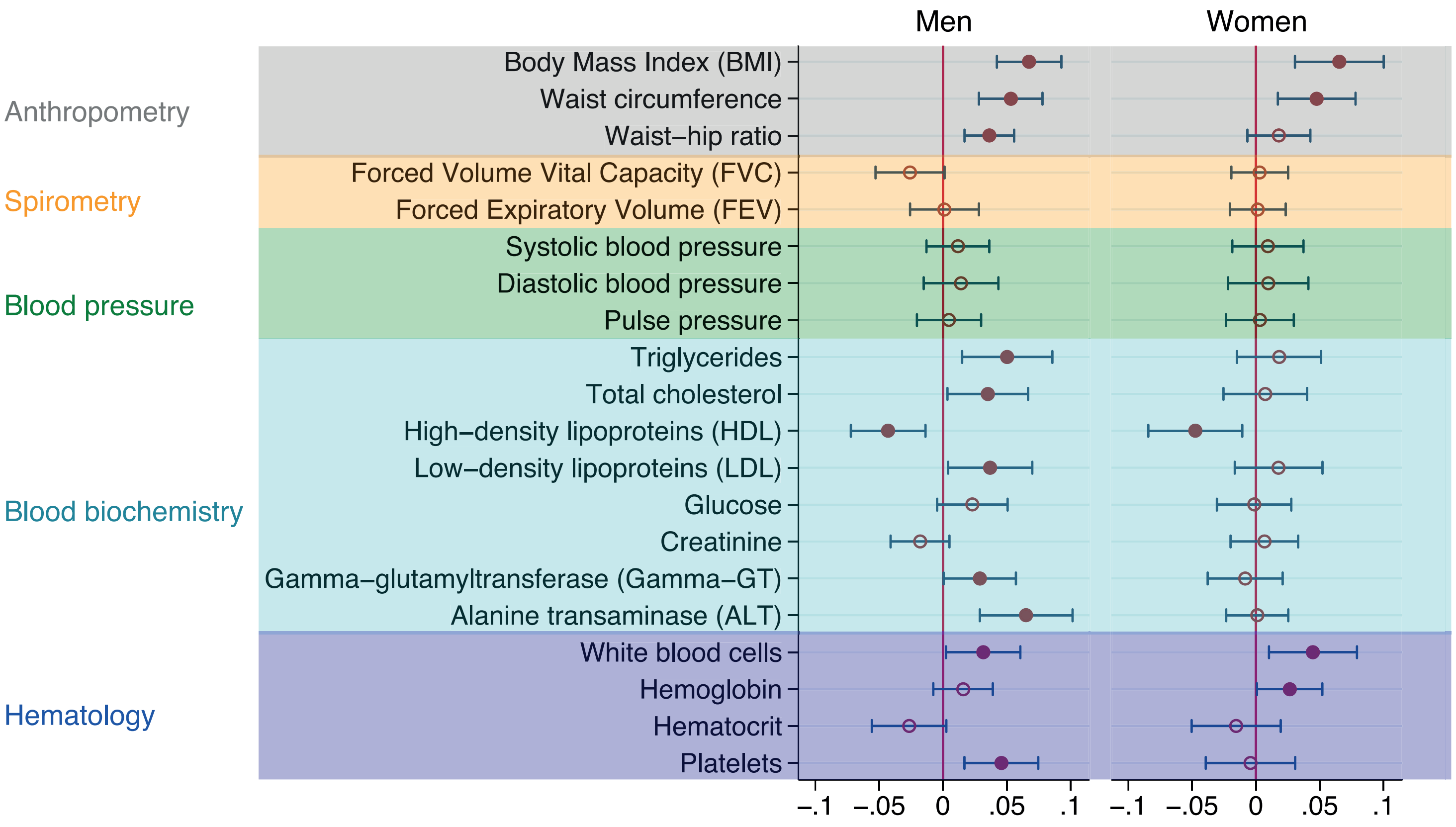

Supplementary figure S2. Results of regression analyses of work stress (ERI ratio>1) and measures from the health examinations among the subsample working full-time, while adjusting for age, socioeconomic position, depressive symptoms, health-related behaviours (physical inactivity, smoking and alcohol consumption) and chronic conditions (cardiovascular disease, endocrine disorders, respiratory disease, osteoarticular arthritis, and cancer). Coefficients estimate the difference on the standardized scale between individuals with work stress compared to those with no work stress.
